# Regenerating human epithelia with cultured stem cells: feeder cells, organoids and beyond

**DOI:** 10.15252/emmm.201708213

**Published:** 2017-12-29

**Authors:** Robert E Hynds, Paola Bonfanti, Sam M Janes

**Affiliations:** ^1^ Lungs for Living Research Centre UCL Respiratory University College London London UK; ^2^ CRUK Lung Cancer Centre of Excellence UCL Cancer Institute University College London London UK; ^3^ The Francis Crick Institute London UK; ^4^ Great Ormond Street Institute of Child Health University College London London UK; ^5^ Institute of Immunity and Transplantation University College London London UK

**Keywords:** 3T3 cells, adult stem cells, cell culture, epithelial cells, organoids, Regenerative Medicine, Stem Cells

## Abstract

More than 40 years ago, Howard Green's laboratory developed a method for long‐term expansion of primary human epidermal keratinocytes by co‐culture with 3T3 mouse embryonic fibroblasts. This was a breakthrough for *in vitro* cultivation of cells from human skin and later for other epithelia: it led to the first stem cell therapy using cultured cells and has vastly increased our understanding of epithelial stem cell biology. In recent years, new methods to expand epithelial cells as three‐dimensional organoids have provided novel means to investigate the functions of these cells in health and disease. Here, we outline the history of stratified epithelial stem cell culture and the application of cultured epithelial cells in clinical therapies. We further discuss the derivation of organoids from other types of epithelia and the challenges that remain for the translation of novel stem cell therapies toward clinical use.

**Glossary**
Clonogenicity: Single epithelial cells have the ability to form clones in culture. Human epithelial cells are heterogeneous in their capacity to form clones. Self‐renewal and growth potential of the colony‐initiating cell can be inferred by secondary culture of individual clones. Secondary cultures generating more than 95% of proliferative clones are “holoclones”, initiated by a stem cell; those generating only small, abortive colonies that terminally differentiate are “paraclones”; finally, “meroclones”, whose secondary cultures contain a mixture of these clonal morphologies, are generated by a progenitor of an intermediate potency.
Feeder cells: 3T3‐J2 murine embryonic fibroblasts provide a supportive *in vitro* environment for the expansion of human stratified epithelial cells. Feeder layers are prepared using mitotically inactivated cells and are gradually outcompeted by growing epithelial cells such that on confluence they form a negligible component of the final product.
Functionality: In generating epithelia for therapy, it is important to distinguish stem cell‐mediated long‐term self‐renewal from short‐term epithelial replacement. Epithelial “bandage” approaches involving transplantation of epithelial cells that were expanded in conditions that do not allow stem cell retention, might be beneficial to stimulate endogenous regeneration but, due to the absence of stem cells, will not themselves maintain the regenerated tissue over the lifetime of the patient.
Long‐term expansion: In optimal culture conditions, epidermal stem cells can be cultured for more than 4 months of continuous culture during which time they undergo over 120 population doublings. Important features of this long‐term expansion are the generation of large numbers of cells for use in therapy (a single epidermal stem cell can generate sufficient cells to generate grafts to cover the whole body surface) and the retention of holoclone‐forming stem cells throughout the culture period. These stem cells underlie the long‐term therapeutic benefit of transplanted cultured epidermis.
Stem cell‐derived organoids: Literature definitions of the term “organoid” differ in scope. The term is often used in a broad sense to capture cell culture systems that are organotypic but here we use it to refer to 3D cultures in which stem cells initiate epithelial tissue formation that is maintained over serial passages.

## Introduction

Primary cell culture of human epithelial cells has been possible since the mid‐1970s, but the ability to establish long‐term cultures has varied depending on which organ cells are isolated from. Nonetheless, research has made considerable progress in understanding the mechanisms by which stem and progenitor cells orchestrate the homeostatic turnover and regenerative potential of adult epithelia. These cells reside within complex niches throughout the body that are composed of differentiated epithelial cells, diverse mesenchymal cells, vasculature, neuronal cells, and surrounding extracellular matrix (ECM).

Cell culture imposes a very different, harsh environment to which epithelial cells must adapt and proliferate extensively without losing their functional potential or entering a senescent state. Defining conditions for expanding primary epithelial cells without immortalization has been a challenge, but, under the correct conditions, cells can undergo more population doublings than they might *in vivo*. Lately, improvements in culture protocols to minimize the loss of clonogenic cells by maintaining a balance between cell proliferation and differentiation have allowed the expansion of sufficient numbers of primary human epithelial cells for diverse applications, including regenerative medicine (De Luca *et al*, 2006), disease modeling (Schweiger & Jensen, 2016), toxicology (Hynds & Giangreco, 2013), and drug discovery (Ranga *et al*, 2014).

In this Review, we focus on the application of cultured epithelial cells in regenerative medicine. In particular, we discuss the 3T3 mouse embryonic fibroblast (MEF) co‐culture system that has enabled stratified epithelial stem cell therapy for clinical use. We further discuss recent advances in 3D organoid culture that allow non‐stratified epithelial cell culture and consider the challenges that these methods face to emulate the translational successes that have used cultured stratified epithelial cells.

## A history of epithelial cell expansion using 3T3 cells

**Figure 1:**
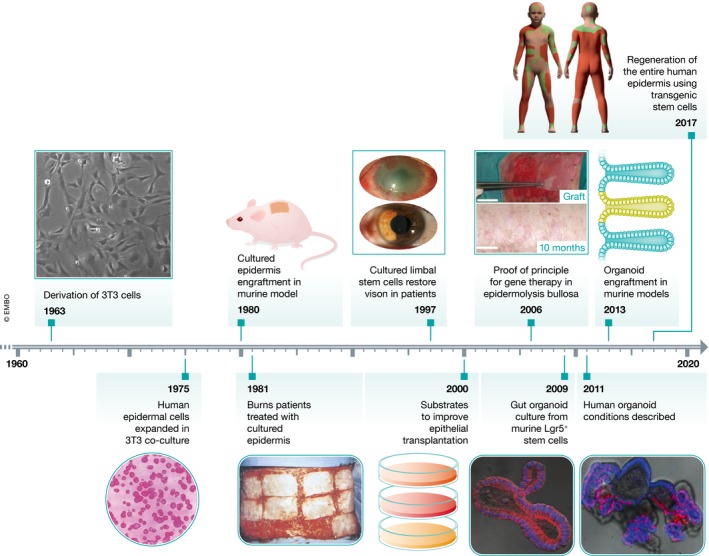
A timeline of advances in long‐term epithelial cell culture Images within this figure have been re‐used with permission from EMBO Press (*EMBO Molecular Medicine*; Droz‐Georget Lathion *et al*, 2015), Massachusetts Medical Society (*The New England Journal of Medicine*, Gallico *et al*, 1984), Dove Medical Press (*Clinical Ophthalmology*, Atallah *et al*, 2016), Nature Publishing Group (*Nature Medicine*, Mavilio *et al*, 2006; *Nature*, Sato *et al*, 2009; Hirsch *et al*, 2017), and Elsevier (*Gastroenterology*, Sato *et al*, 2011a).

3T3 cells were first isolated in Howard Green's laboratory in the early 1960s as part of his experiments to better control the process of cell line derivation (Fig 1). Mouse embryonic cells were plated to select for adherent cells after embryo disaggregation and then expanded for 2–3 days until confluence. Following this transfer or “passage”, culture conditions—inoculation densities and transfer frequency—were systematically varied to study the growth characteristics of murine fibroblasts (Todaro & Green, 1963). Higher inoculation densities increased the possibility of routine culture establishment, but one cell line was established from a low‐density culture. “3T3” is thus an abbreviation of this culture protocol—a 3‐day transfer period with 3 × 10^5^ cells plated at each transfer. In common with MEF cultures established using other protocols, 3T3 cells developed altered chromosome number and gross karyotypic abnormalities, but were contact‐inhibited upon cell confluence. Initially, the cells were used in studies of viral transformation, as loss of contact inhibition can be easily identified in a confluent monolayer (Todaro & Green, 1966).

Reproduction of the 3T3 protocol in other laboratories led to isolation of independent 3T3 cell lines from BALB/C and NIH Swiss mouse strains, giving rise to BALB/3T3 and NIH/3T3 lines, respectively (Aaronson & Todaro, 1968; Jainchill *et al*, 1969). These lines differ from the original 3T3 line as they are less contact‐inhibited and more susceptible to viral transformation. Other subclones of Green's 3T3 cells were selected for the presence of lipid accumulation. Upon cell confluence, these cells accumulate triglyceride lipids in cytoplasmic droplets and generate mature fat pads after injection into athymic mice (Green & Kehinde, 1974, 1979; Green & Meuth, 1974). These cells, known as 3T3‐L1, are now widely used to model adipogenesis.

With regard to epithelial cell expansion *in vitro*, a key breakthrough was made during attempts to derive primary cell cultures from murine teratomas. When cultured on plastic, these tissues gave rise to fibroblasts with few, poorly proliferative epithelial cells. However, co‐culture with mitotically inactivated 3T3 cells reduced fibroblast contamination and enabled serial expansion of epithelial cells resembling keratinocytes (Rheinwald & Green, 1975a). Application of this protocol to human epidermal keratinocytes, which had until then proven impossible to expand substantially in culture, created progressively growing, strongly adhesive colonies of epithelial cells that pushed away the surrounding feeder cells (Rheinwald & Green, 1975b). These colonies are genuine 3D structures that can display partial stratification, with cells resembling basal cells in contact with the culture dish.

Both the feeder cells and the culture medium were further optimized for human keratinocyte culture, which now uses 3T3‐J2 cells, a subclone of Green's 3T3 line that are more supportive of keratinocyte cultures (Rheinwald, 1989). Initially, co‐cultures were performed in medium consisting of Dulbecco's modified Eagle's medium (DMEM) and Ham's F12 in a 3:1 ratio with added fetal bovine serum (FBS) and hydrocortisone. Later refinements saw the addition of adenine (Peehl & Ham, 1980), cholera toxin (Green, 1978; Okada *et al*, 1982), insulin (Tsao *et al*, 1982), triiodothyronine (Maciag *et al*, 1981), and epidermal growth factor (Rheinwald & Green, 1977). 3T3 co‐culture in supplemented FAD (F12, adenine, and DMEM) medium allowed the long‐term expansion of human epithelial cells for the first time.

Importantly, long‐term expansion of epidermal cells in culture is enabled by the maintenance of epidermal stem cells; this was shown by seminal experiments that allowed the retrospective identification of stem cells by analyzing the differential growth capacity of colony‐forming human epidermal cells *in vitro* (Barrandon & Green, 1987). When individual colonies formed from a single cell are re‐plated in secondary cultures, they can be classified into three different clonal types: the “holoclone” has the greatest expansion capacity as at least 95% of the colonies in secondary cultures are large and contain small, highly proliferative cells; the “paraclone” gives rise only to small colonies of cells that undergo terminal differentiation within a few doublings (< 15); finally, the “meroclone” represents an intermediate stage between holoclones and paraclones that contains both types of colonies (Barrandon & Green, 1987). Cells that form holoclones are the epidermal stem cells that are able to reconstitute a functional epidermis lasting for a lifetime in the treatment of full‐thickness burns (Pellegrini *et al*, 1999). The number of holoclones *in vivo* is affected by aging, whereas loss of stemness in culture may occur by clonal conversion—from holoclones, through meroclones to paraclones—during which growth potential progressively decreases and telomere‐independent senescence takes hold (Barrandon *et al*, 2012).

## Clinical translation of epidermal and corneal stem cells

Endogenous regeneration mediated by adult stem cells is not always capable to completely repair damaged tissue. For example, full‐thickness epidermal wounds larger than 4 cm in diameter do not heal appropriately without medical intervention (MacNeil, 2007). Current therapies involve repair with split‐thickness autografts of epidermis and a thin layer of dermis from a non‐affected body site. However, this approach is poorly suited for extensive burns and increases the risk of sepsis. The clinical application of autologous stem cells expanded *ex vivo* has addressed this problem. By the early 1980s, pre‐clinical work demonstrated that epithelial sheets could be generated by culturing keratinocyte colonies to confluence and detaching them using enzymes that target cell–substrate but not cell–cell junctions, such as dispase (Banks‐Schlegel & Green, 1980) or thermolysin (Germain *et al*, 1993). Engraftment of these cultured epidermal autografts (CEAs) on wound beds in immune‐compromised mice was robust, and the histological morphology of cultured human epidermis remained intact for several months after transplantation (Banks‐Schlegel & Green, 1980).

Initially, two patients received CEAs in combination with conventional autografts to treat extensive third‐degree burns (O'Connor *et al*, 1981). Large‐scale production followed and two patients with third‐degree burns of more than 80% of the total skin surface also received CEAs (Gallico *et al*, 1984). Engraftment was successful, and around 50% of the life‐saving epithelial regeneration was mediated by cultured cells. Overall, 60–100% of grafts take (Gallico *et al*, 1984; Pellegrini *et al*, 1999), but the success rate is highly variable between individual patients (De Luca *et al*, 2006) and grafts can fail owing to suboptimal wound bed preparation and infection.

An improvement to the CEA protocol has involved culturing keratinocytes on alternative substrates as dispase‐mediated detachment shrinks the epithelial surface area by 50% and generates fragile epithelial sheets that can only be used during a short window around the time of cell confluence. These alternative substrates include fibrin (Pellegrini *et al*, 1999; Ronfard *et al*, 2000), an insoluble protein mesh that is formed during normal wound healing, ECM proteins (Myers *et al*, 1997; Horch *et al*, 2000), and chemically defined surfaces (Zhu *et al*, 2005), but preservation of epidermal stem cells has only been demonstrated on tissue‐culture plastic and fibrin substrates. These substrates have multiple advantages as they remove the need for enzymatic dissociation, retain ECM proteins produced by epithelial cells, and are more easily manipulated during surgery. Crucially, substrate‐enabled CEAs can be transplanted subconfluent, which permits a more flexible time window for transplantation. The performance of CEAs was also appreciably improved by the use of cadaveric skin allografts, which adhere to the wound bed; the epidermis can be mechanically removed later for CEA engraftment (Cuono *et al*, 1986). This reduces the risk of infection and therefore promotes successful engraftment by maintaining a skin barrier prior to CEA transplantation and also provides a surgically prepared wound bed of allodermis.

Clinical experience and post‐treatment analysis showed that CEAs are safe and that the morphological features of epidermis return, albeit over variable timeframes: scar tissue in the dermal layer takes up to 5 years to resolve, whereas Langerhans cells are present within 6 months and melanocytes within a year. Normal interfollicular epidermal histological appearance is maintained for many years post‐transplantation (Compton *et al*, 1989; Odessey, 1992). In this way, 3T3 co‐culture has benefitted thousands of patients worldwide (Watt, 2014). However, epidermal appendages such as hair follicles, sebaceous glands, and sweat glands are not currently regenerated in CEA skin, although progress has been made toward understanding the molecular mechanisms that govern their development (Lu *et al*, 2016; Chacon‐Martinez *et al*, 2017; Lei *et al*, 2017) and designing methods to incorporate these into clinical transplants (Higgins *et al*, 2013).

Notably, CEA transplantation has revealed novel insights into the extent of epidermal cell plasticity. Palm and sole epidermis for instance is characterized by a thickened stratum corneum and cytokeratin‐9 (KRT9) expression, which are not found in epidermis from other anatomical sites. When palm‐derived CEAs were grafted onto a patient's upper leg, they retained memory of their site of origin (Mavilio *et al*, 2006). Similarly, cultured oral keratinocytes from the palate preserve donor site characteristics after transplantation (De Luca *et al*, 1990). It appears that keratinocyte location specificity is cell intrinsic and not lost during culture or re‐wired by the microenvironment following transplantation. The epigenetic mechanisms underlying this phenomenon are not currently understood.

More recently, CEAs have enabled epithelial gene therapy in patients with junctional epidermolysis bullosa (JEB), a blistering disease in which epithelia are inadequately anchored to the basement membrane owing to mutations in genes encoding laminin 332 (*LAMA3*,* LAMB3,* and *LAMC2*). Autologous epidermal keratinocytes expanded in culture and retrovirally transduced with full‐length *LAMB3* have been successfully engrafted as sheets onto surgically prepared wound beds (Mavilio *et al*, 2006; Hirsch *et al*, 2017). LAMβ3 mRNA was detectable in grafts and expression of the transgene allowed proper keratinocyte‐basement membrane interaction via integrin α6β4. Healthy epithelium containing a normal number of ΔNP63 isoform α‐positive keratinocyte basal cells was regenerated in transplanted areas (De Rosa *et al*, 2014). In a very recent report, more than 80% of a patient's total epidermal surface (Hirsch *et al*, 2017) was transplanted after *in vitro* gene correction. This is a landmark successful *ex vivo* gene therapy for a genetic disease of the epithelium. Nevertheless, these gene therapy studies face the risk that more than one‐third of retroviral integration sites can fall within transcriptionally active genes; however, since long‐term regeneration depends only on a small number of stem cells, the significance of deleterious insertion sites might be overstated, especially when balanced with absence of treatment that, in cases like this, invariably results in the patient's death. Together, these studies demonstrate that cultured epithelial cells can engraft and contribute to long‐term regeneration providing that they are given the correct cues for self‐renewal *in vitro*. Future application of gene editing technologies, such as adeno‐associated viral vectors (AAVs; Melo *et al*, 2014) or high‐fidelity CRISPR‐Cas9 systems (Kleinstiver *et al*, 2016), could be safe and effective means to generate gene‐corrected, clonally derived CEAs for transplantation but are not yet in clinical use (Droz‐Georget Lathion *et al*, 2015).

3T3 co‐culture is effective for culturing other stratified squamous epithelia (Romagnoli *et al*, 1990), most notably for the generation of corneal epithelium to treat severe ocular burns (Atallah *et al*, 2016). Owing to the anatomical location of the stem cells responsible for maintaining the clear corneal epithelium in the limbus (Cotsarelis *et al*, 1989)—the border area between cornea and conjunctiva—ocular burns can deplete the stem/progenitor population. In their absence, conjunctival cells migrate across the cornea causing a connective coverage that leads to complete loss of vision. Limbal restoration is therefore essential for successful therapy. For patients with minimal residual limbal tissue or an uninjured eye, limbal stem cells expanded in culture restore vision upon transplantation (Pellegrini *et al*, 1997; Rama *et al*, 2010). To date, several hundred patients have been treated and, in an Italian cohort, vision was successfully restored in 78% of cases (De Luca *et al*, 2006).

The most important lesson from these epithelial therapies is that of stem cell retention during culture. In CEAs and limbal transplants alike, the retention of stem cells, rather than the histological appearance of the epithelium *in vitro*, is the critical indicator for graft success (Pellegrini *et al*, 1999). If culture is suboptimal, and stem cells lost by clonal conversion, grafts generate an atrophic, fragile epithelium that eventually fails due to the ever‐decreasing ability of transplanted cells to maintain epithelial integrity (Pellegrini *et al*, 2013). Importantly, a retroviral integration study of genetically defined clones in engrafted epidermis in the recent clinical application of CEA technology for JEB clearly demonstrates that only a small number of transplanted stem cells maintain long‐term regeneration *in vivo* (Hirsch *et al*, 2017).

Yet, the retrospective method for identifying stem cells *in vitro* by clonal culture is not well suited for monitoring stem cell maintenance in cultures intended for clinical application. As an alternative, expression levels of the transcription factor P63 are a more direct method to assess stem cell content, at least in limbal epithelial cell cultures (Pellegrini *et al*, 2001; Di Iorio *et al*, 2005). In mice, *P63*‐knockouts are born with stratified epithelia consisting of differentiated cell types, but their maintenance of basal epithelial stem cells is deficient (Yang *et al*, 1999). There are at least 10 P63 isoforms classed as either transactivating (TP63) or N‐terminal truncated isoforms (ΔNP63) each with different C‐termini (α, β, γ, δ, and ε; Mangiulli *et al*, 2009). The ΔNP63 isoform α is enriched in stem cells and is essential for their retention (Blanpain & Fuchs, 2007; Senoo *et al*, 2007). Holoclone formation and ΔNP63 expression levels could both be used for clinical quality control (Pellegrini *et al*, 2013). Recently, a non‐invasive, time‐lapse imaging method was developed which determines epidermal stemness during colony growth through analysis of cell movement; colonies with collective motion are more likely to be holoclones (Nanba *et al*, 2015).

## Recreating a niche *in vitro*

### 3T3 cells: a mechanistic enigma

3T3 co‐culture remains the gold standard for stem cell expansion in many settings as feeder‐ and serum‐free media, which often replace these with crude bovine pituitary extract (Peehl & Ham, 1980), stimulate proliferation for only short periods. 3T3 co‐culture starts with producing feeder layers by inactivating 3T3 cells with ~60 gray (Gy) ionizing (gamma) radiation, which causes direct DNA damage through double‐stranded breaks and the generation of free radicals that interact with DNA, or by treating 3T3 cells with mitomycin C, which covalently cross‐links double‐stranded DNA and prevents the separation of DNA strands during replication. Both methods cause irreversible cell cycle arrest and altered cellular metabolism; the inactivated state might be required in its own right for epithelial cell support (Palechor‐Ceron *et al*, 2013).

Nonetheless, the contribution of 3T3 cells in co‐culture is poorly understood, which explains the difficulties in producing defined media that fully recapitulate their growth‐supportive effects. This effect is not simply caused by an increase in the overall density of cells and is fibroblast‐specific. Indeed, other strains of MEFs or even virally transformed 3T3 cells are not supportive of keratinocyte expansion to the same extent (Green *et al*, 1977). Although conditioned medium from 3T3 cells also cannot fully reproduce the effect of co‐culture, diffusible factors likely mediate some of their key effects as separation of epithelial and feeder cells using transwell membranes allows colony formation (Palechor‐Ceron *et al*, 2013). Instead, a constant supply of feeder cell factors, which cannot be recreated using conditioned medium, might be necessary. Further work is required to assess the effect of cell–cell contact on clonogenicity.

Previous work has also stressed the importance of reciprocal signaling between epithelial and feeder cells for optimal culture conditions. The receptor tyrosine kinase response of epithelial cells treated with 3T3 fibroblast‐conditioned medium is surprisingly modest (R.E. Hynds, P. Bonfanti, S.M. Janes, unpublished data), but cultured keratinocytes constitutively produce interleukin‐1 (IL‐1), which causes c‐JUN‐dependent production of proliferation‐enhancing growth factors by 3T3 cells, including keratinocyte growth factor (KGF), granulocyte–macrophage colony‐stimulating factor (GM‐CSF), and hepatocyte growth factor (HGF; Szabowski *et al*, 2000; Schnickmann *et al*, 2009). Insulin‐like growth factors (IGFs) have also been identified as relevant 3T3 cell factors since IGF inhibition in keratinocytes or replacing 3T3‐J2 cells with BALB/3T3 cells, which do not produce IGFs, reduces epithelial proliferation (Barreca *et al*, 1992). The secretion of IGF2 in 3T3‐J2 cells appears to be due to their higher expression of the Wnt pathway antagonist Dact1, which suppresses transforming growth factor‐β2 (TGFβ2) production in response to epithelial‐derived Wnt signals (Suzuki & Senoo, 2015). This synergy between epithelial cells and fibroblasts, along with the fact that xenogeneic proteins bind to and activate receptors on human epithelial cells with different efficiency to species‐relevant proteins, has complicated studies to unravel the mechanistic basis of 3T3 co‐culture.

A recent protocol to improve multiple feeder‐free stratified epithelial cell culture systems uses A83‐01 and DMH‐1, inhibitors that target activin‐like kinase (ALK) receptors in the TGFβ and BMP signaling cascades, respectively (Mou *et al*, 2016). Interestingly, this raises the possibility that the role of feeder cells might be the elimination of factors that are detrimental for epithelial cell propagation, such as TGFβ in serum or produced by epithelial cells, rather than to provide feeder factors. Nevertheless, the ability of feeder‐free systems to preserve stem cells has not been demonstrated and so, while our understanding of how 3T3‐J2 cells support diverse stratified epithelial cells remains limited, this system remains the gold standard for epidermal and limbal regenerative medicine.

### Development of organoid cultures

**Figure 2:**
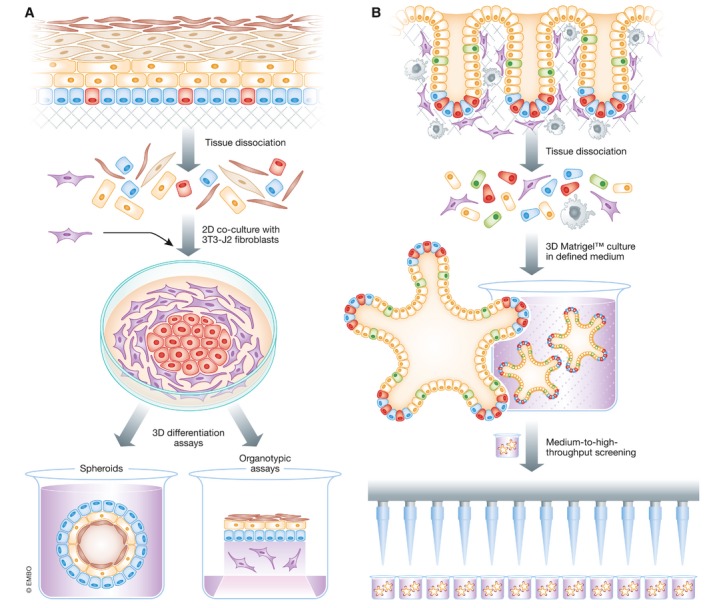
Strategies of adult stem cell‐based epithelial cell culture (A) Pathway for isolating human epithelial cells in 3T3 co‐culture and methods for their “re‐differentiation” in organotypic assays. (B) Pathway for isolating human epithelial cells as 3D organoids.

The term organoid has been applied to organotypic systems that more closely represent *in vivo* tissues (Fig 2) than 2D cultures which often lose key aspects of tissue physiology (Sasai, 2013). The loss of the complex microenvironment *ex vivo* leads to less representative cellular organization or even an inability to initiate cell cultures. By seeding cells into a 3D matrix in the presence of mesenchyme (Ootani *et al*, 2009) or in medium containing epidermal growth factor (EGF), Noggin and a Wnt pathway potentiator, R‐spondin 1 (Sato *et al*, 2009), epithelial cells can be expanded long term even from simple epithelia that were traditionally hard to maintain in cell culture. By way of example, the dependence of LGR5^+^ gastrointestinal stem cells on Wnt pathway activation has allowed the formulation of media to expand organoids from the human gastrointestinal tract that retain the crypt–villus architecture of the native intestine (Sato *et al*, 2011a). Subsequently, self‐renewing organoids have been derived from organs, including the prostate, liver, and pancreas, expanding the repertoire of human epithelial tissues from which primary culture is possible (Clevers, 2016; Kretzschmar & Clevers, 2016).

There are differences between epithelia that can be cultured as 2D 3T3 co‐culture and as 3D organoids. 3T3 cells support long‐term P63^+^ stratified epithelial stem cell expansion, whereas there are currently no reports of organoid culture methods to expand stratified epithelia long‐term, suggesting that these cells might have different self‐renewal requirements. Wnt signaling, an essential requirement for organoid propagation, is unlikely to be the basis of 3T3 cell keratinocyte support as 3T3‐J2 cells express comparable levels of Wnt proteins to other MEFs that do not support expansion (Suzuki & Senoo, 2015). Furthermore, Green's protocol has remained relatively unchanged between different stratified epithelia, but the growth requirements for different human simple epithelial organoids are more diverse.

Organoid cultures have yielded insight into the cellular mechanisms of epithelial homeostasis and helped to elucidate specific niche factors. For example, small intestinal Paneth cells, an antimicrobial‐secreting cell population adjacent to LGR5^+^ stem cells, produce EGF receptor ligands, Notch ligands, and WNT3, all of which are essential for stem cell maintenance in small intestinal organoids (Sato *et al*, 2011b). c‐KIT^+^/REG4‐expressing deep crypt secretory cells function in a similar manner in the colonic epithelium (Rothenberg *et al*, 2012; Sasaki *et al*, 2016). This highlights the importance of both secreted and membrane‐bound intraepithelial signals as niche components, in addition to more commonly studied mesenchymal–epithelial pathways (Farin *et al*, 2016).

Furthermore, the location specificity of LGR5^+^ gastrointestinal tract stem cells is retained during organoid culture (Middendorp *et al*, 2014). Consistent with the finding that epidermal stem cells retain memory of their donor site in clinical studies (Mavilio *et al*, 2006), murine small intestinal organoids transplanted into the murine colon retain transcriptional hallmarks of small intestinal epithelium 4 weeks after engraftment (Fukuda *et al*, 2014). Expression of colonic epithelium‐specific markers after transplantation of fetal small intestinal organoids suggests that these cells might be more plastic (Fordham *et al*, 2013). Overall, however, existing data do not support the notion that adult stem cell expansion protocols “reprogram” cells to greater potency but rather maintain their native identity and functionality.

**Figure 3:**
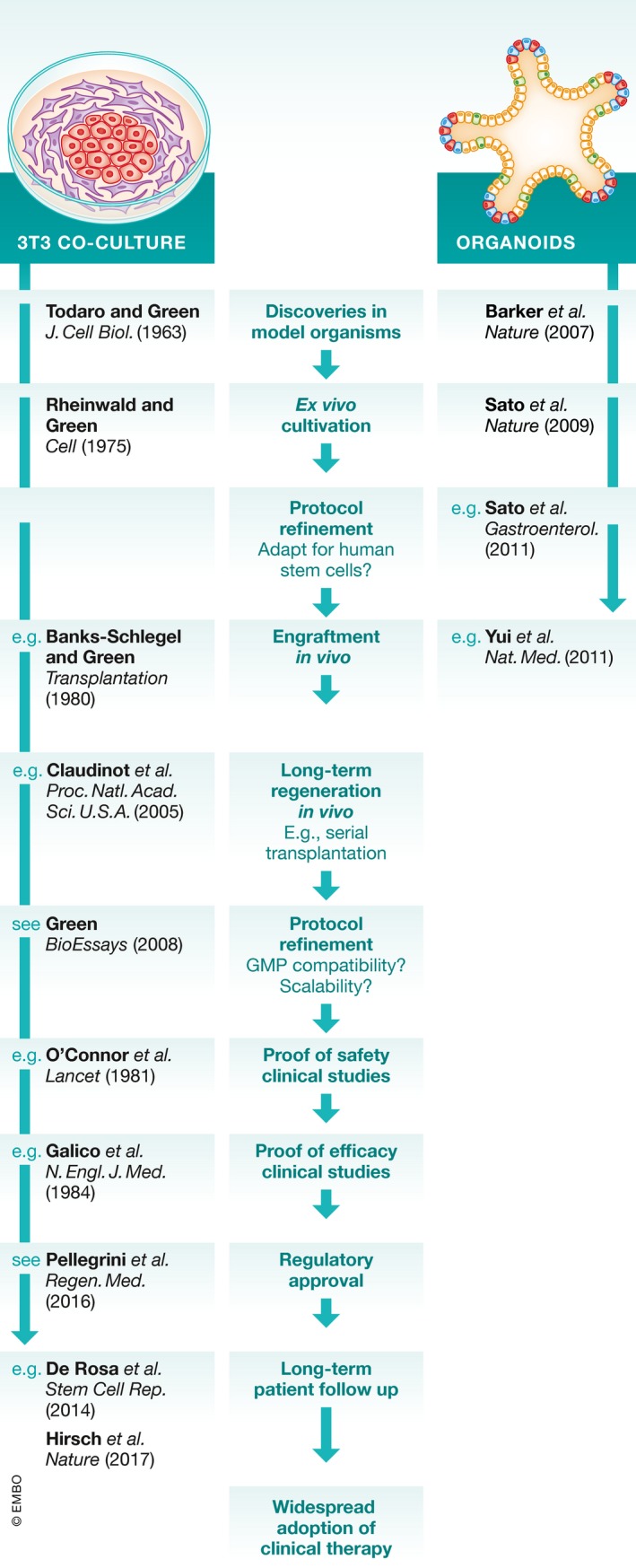
Milestones in regenerative medicine using cultured epithelial stem cells

Despite the rapid progress in organoid technologies, the field is at a relatively early stage of development in terms of clinical use (Fig 3); in particular, questions remain as to whether these cells can maintain tissue homeostasis after transplantation. Experiments involving transplantation of murine colonic organoids in an acute colitis model show promising results: organoids derived from a single stem cell were capable of engrafting, surviving, and contributing to histologically normal epithelium for more than 25 weeks (Yui *et al*, 2012). Data demonstrating engraftment of human organoids in orthotopic murine transplantation experiments are currently limited to human hepatic organoids in acute liver injury (Huch *et al*, 2015). Transplanted cells differentiated to express hepatocyte markers and human albumin and alpha‐1‐antitrypsin could be detected in the blood of successfully transplanted animals after 120 days. However, the functionality of the engrafted cells, including their contribution to the hepatic stem cell pool, was not analyzed.

These initial transplantation experiments have also revealed practical hurdles for regenerative applications as vast numbers of organoid‐derived cells have enabled only very modest engraftment rates. Experimentally, this might be addressed by improving the reproducibility between and within organoid cultures, by enhancing delivery methods, and by upscaling organoid cultures for medical applications, particularly within clinically useful timeframes. Overall, the evidence for using organoids as a cell source in translational applications falls short of demonstrating fully functional epithelia and further pre‐clinical animal models should be advanced.

### Dependence of cultured epithelial cells on xenogeneic factors

A commonality between 3T3 co‐culture and organoid culture systems is that the gold standard protocols for stem cell culture depend on murine‐derived products. Attempts to replace 3T3 cells with human feeder cells and/or defined media have been largely unsuccessful in terms of stem cell maintenance, while the regulatory environment for cell therapies has changed substantially. Early grafts from 3T3 co‐culture were sanctioned through collective agreement about the scientific rationale by a small number of clinicians (Green, 2008). More recently, regulatory bodies in the USA and Europe have classified tissues based on 3T3 co‐culture as xenografts. The use of murine cells and bovine serum raises concerns about the transmission of infectious agents. It was also suggested that co‐culture of human embryonic stem cells (hESCs) with murine feeder cells leads the former to incorporate a potentially immunogenic non‐human sialic acid, Neu5Gc (Martin *et al*, 2005). However, it was later shown that human sera do not reduce hESC viability substantially and that minor effects are independent of cellular Neu5Gc content (Cerdan *et al*, 2006). Notably, these concerns are unsupported by the long‐term engraftment and survival of transplanted epithelial cells in patients. While adverse events are reported, these primarily relate to the preparation of the graft site and the quality of epithelial cells transplanted, rather than the presence of 3T3 cells, which only account for 0–2% of cells in the final CEA product (Ronfard *et al*, 2000). Importantly, no tumor development has been reported in any patients even after follow‐up over several decades. CEAs have received regulatory approval in a number of countries including the USA, Japan, and Korea. In Europe, limbal stem cell therapy was recently approved contingent upon the generation of a well‐characterized 3T3‐J2 master cell bank in which all feeder cells are infection‐free and cultured in accordance with Good Manufacturing Practice (Pellegrini *et al*, 2014).

To make matters more complicated, the interactions between epithelial cells and the ECM secreted by murine feeder cells in 3T3 co‐culture are poorly understood. The complex ECM is known to contain fibronectin, laminin, and collagen types I, IV, and V (Alitalo *et al*, 1982), but their relative importance for epithelial stem cell retention is not known. Laminin is an interesting candidate: a recent feeder‐free protocol employs laminin‐rich conditioned medium from 804G rat bladder carcinoma cells to deposit a substrate for epithelial cell expansion (Mou *et al*, 2016) and the common organoid ECM from murine sarcoma‐derived Matrigel contains predominantly laminin, collagen IV, and nidogen‐1, but it also includes more than 1,000 other proteins with variability between batches (Hughes *et al*, 2010). Laminin 511 has been used as a matrix for feeder‐free growth of pluripotent (Nakagawa *et al*, 2014) and epithelial cells (Polisetti *et al*, 2017) but, overall, the physiological presentation of ECM secreted by cells seems to be more efficient compared with systems that use recombinant ECM proteins.

Replacing Matrigel with a better‐defined product will be critical for future translation of human organoids for clinical applications. The potential use of bovine collagen I as a substitute (Jabaji *et al*, 2014) might address some concerns, but the conditions for complete organoid differentiation in collagen gels have not been described and their effects on the long‐term maintenance of stem cells are unclear. Recent studies have begun to pave the way for Matrigel replacement with synthetic polymers: one elegant study sets out to determine minimal *in vitro* ECM niche requirements using a bottom‐up bioengineering approach (Gjorevski *et al*, 2016). LGR5^+^ stem cells could be maintained in a synthetic RGD‐functionalized polyethylene glycol (PEG) hydrogel, but these organoids lacked differentiated cell types. Hydrogel stiffness, communicated through YAP signaling, was the key determinant of stem cell retention and differentiation: an initially stiff matrix was required for stem cell expansion and subsequent matrix softening allowed differentiated cell types to emerge. The dynamic biomechanical properties of Matrigel therefore likely support both stem cell maintenance and organoid differentiation. A second study, investigating an alternative synthetic approach based on PEG‐4MAL macromer‐based hydrogels, also emphasized the importance of polymer mechanical properties and showed that these could expand pluripotent cell‐derived intestinal organoids that could engraft *in vivo* (Cruz‐Acuna *et al*, 2017). In such studies, full biocompatibility and rate of polymer degradation should be carefully analyzed before clinical translation.

### Maintaining stem cells in culture

As discussed above, the abundance of stem cells, or holoclones, in transplanted skin and corneal grafts determines the long‐term survival of the graft. It might therefore be beneficial to use products with a high proportion of stem cells. In epidermal 3T3 co‐cultures, partial differentiation of colonies occurs during their expansion, while the stem cell population is retained by balanced self‐renewal of stem cells. Isolation of these cells in the presence of Rho‐associated protein kinase inhibition (ROCKi) can improve the number of cells expanded in culture, potentially including a greater number of stem cells (Chapman *et al*, 2010; Terunuma *et al*, 2010), likely by decreasing the extent of anoikis in the initial cell suspensions (Watanabe *et al*, 2007). Continuous ROCKi or mammalian target of rapamycin (mTOR) inhibition using rapamycin (Iglesias‐Bartolome *et al*, 2012) also increase epithelial cell proliferation and reduce terminal differentiation (McMullan *et al*, 2003; Nanba *et al*, 2013) but, despite evidence that cells expanded with ROCKi can differentiate appropriately *in vivo* (Butler *et al*, 2016), the capacity of cells grown in these conditions to contribute to long‐term organ homeostasis after transplantation has not yet been shown. This stands in contrast to Green's protocol, which has clearly demonstrated this ability in multiple epithelial cell types.

ROCKi is also used during 3D organoid protocols to improve cell viability during isolation and transfer but does not significantly alter the cellular composition of organoids. Multiple groups have explored strategies to preferentially expand LGR5^+^ stem cells, rather than their differentiated progeny, in 3D organoid cultures. Addition of the Wnt pathway activator CHIR99021 and the histone deacetylase inhibitor valproic acid to culture medium favors expansion of murine Lgr5^+^ intestinal stem cells over differentiated cell types in organoid culture (Yin *et al*, 2014). Interestingly, the combination of 3T3 co‐culture and medium with niche factors used in organoid culture allows long‐term, pure expansion of genome‐stable human gastrointestinal stem cells in 2D culture (Wang *et al*, 2015), suggesting that apico‐basal polarity generated in 3D organoid cultures is key to the emergence of differentiated cell types. It will be important to establish in pre‐clinical transplantation models whether stem cell‐enriched transplants outperform those that contain differentiated cell types.

## Future perspectives

Highly expandable cultures containing epithelial stem cells from a variety of organs can be established in co‐culture with 3T3 cells. These co‐cultures, in combination with organotypic differentiation assays, already offer a great tool for pre‐clinical research, but the use of murine feeder cells is a limitation for their routine use in therapy. However, since stem cell retention is essential for long‐term integration of cultured epithelia, the premature translation of therapies that attempt to replace components of the culture system without clear evidence that they avoid the loss of stem cells should be cautioned against. Alternatively, trials should have a clear rationale for the use of cells that will not support long‐term tissue homeostasis. Unraveling the mechanisms involved in 3T3 support of stratified epithelial stem cells has proved difficult, but it is yielding more knowledge about basic stem cell biology. The determination of essential feeder factors and the production of defined growth conditions for pluripotent stem cells give hope that it will be possible to replace murine feeder cells for epithelial stem cell culture in the future (Nakagawa *et al*, 2014).

There is much excitement in regenerative medicine about the potential clinical use of iPS cells and much progress has been made toward differentiating these into multiple organ‐specific lineages, including epithelium. Yet, there remain greater hurdles to the use of iPS‐derived organoids compared to tissue‐specific, stem cell‐derived cultures, including mutational burden, culture duration, differentiation efficacy, epigenetic changes, and ultimate functionality. The impact of culture on the genome of cells is a particular concern. While the absence of any clinically apparent mutations in patients receiving cultured epithelial cell grafts to date is encouraging, rare genomic alterations could still occur during expansion. Initial data suggest that small numbers of *de novo* mutations are introduced during organoid culture, but this nevertheless compares favorably to the number of mutations that are introduced during induced pluripotent stem (iPS) cell culture (Huch *et al*, 2015). For these reasons, adult epithelial cells remain the first choice for clinical application, but iPS options should be explored if the use of adult stem cells is not feasible (Bilousova & Roop, 2014). This might include simple epithelia, cases where adult stem cells have been destroyed by injury—such as severe bilateral burns of the cornea—or if whole tissue substitutes (e.g., full‐thickness skin) can be developed (Workman *et al*, 2017). Overall, the cell culture advances described here raise the prospect that the translational success achieved for skin and cornea could be repeated in further epithelial tissues.

## Conflict of interest

The authors declare that they have no conflict of interest.

**Pending issues**

- **Safety Issues.** Culture systems for stratified epithelial cells that are not dependent on mouse 3T3 feeder cells would be an important step. However, it is crucial that these are demonstrated to have the equivalent capacity to maintain epithelial stem cells with long‐term *in vivo* regenerative capacity.
- **Clinical relevance of organoids.** The application of organoid‐derived epithelial therapies will require the development of clinical‐grade matrices, scaled‐up culture platforms, and efficient transplantation methods.
- **Barriers to widespread implementation.** The use of autologous adult stem cells in personalized medicines raises issues around implementation in large patient cohorts. Currently, the manufacture of such products is expensive and limited to very specialized centers.
